# Isolation and Biosynthetic Analysis of Haliamide, a New PKS-NRPS Hybrid Metabolite from the Marine Myxobacterium *Haliangium ochraceum*

**DOI:** 10.3390/molecules21010059

**Published:** 2016-01-06

**Authors:** Yuwei Sun, Tomohiko Tomura, Junichi Sato, Takashi Iizuka, Ryosuke Fudou, Makoto Ojika

**Affiliations:** 1 Graduate School of Bioagricultural Sciences, Nagoya University, Furo-cho, Chikusa-ku, Nagoya 464-8601, Japan; ch3cook@gmail.com (Y.S.); tomura.tomohiko@a.mbox.nagoya-u.ac.jp (T.T.); john-smith-11@live.jp (J.S.); 2 Institute for Innovation, Ajinomoto Co., Inc., Kawasaki, Kanagawa 210-8681, Japan; takashi_iizuka@ajinomoto.com; 3 R & D Planning Department, Ajinomoto Co., Inc., Chuo-ku, Tokyo 104-8315, Japan; ryosuke_fudou@ajinomoto.com

**Keywords:** marine myxobacterium, *Haliangium ochraceum*, haliamide, polyketide, biosynthesis

## Abstract

Myxobacteria of marine origin are rare and hard-to-culture microorganisms, but they genetically harbor high potential to produce novel antibiotics. An extensive investigation on the secondary metabolome of the unique marine myxobacterium *Haliangium ochraceum* SMP-2 led to the isolation of a new polyketide-nonribosomal peptide hybrid product, haliamide (**1**). Its structure was elucidated by spectroscopic analyses including NMR and HR-MS. Haliamide (**1**) showed cytotoxicity against HeLa-S3 cells with IC_50_ of 12 μM. Feeding experiments were performed to identify the biosynthetic building blocks of **1**, revealing one benzoate, one alanine, two propionates, one acetate and one acetate-derived terminal methylene. The biosynthetic gene cluster of haliamide (*hla*, 21.7 kbp) was characterized through the genome mining of the producer, allowing us to establish a model for the haliamide biosynthesis. The sulfotransferase (ST)-thioesterase (TE) domains encoded in *hlaB* appears to be responsible for the terminal alkene formation via decarboxylation.

## 1. Introduction

Myxobacteria are unique Gram-negative bacteria characterized by gliding on solid surfaces and formation of multicellular fruiting body [1,2,3]. Although long been regarded as terrestrial microorganisms, several halophilic myxobacterial strains have been noticed recently [4,5,6,7,8] and found to be excellent producers of novel secondary metabolites, such as polyketides (PKs), non-ribosomal peptides (NRPs) and their hybrids. A recent report suggested that the polyketide synthase (PKS) gene sequences in marine-derived myxobacterial isolates are highly novel (all of the sequences were less than 70% similar to the known ones) [9]. However, studies of the secondary metabolites of marine myxobacteria have been hampered by unfavorable factors such as difficulties in the isolation from environment, slow growth rates, strong tendency of cell aggregation and poor metabolite productivity. Up to now, there are a few groups of antibiotics that have been identified from the marine environment, including haliangicins [10,11,12], miuraenamides [13,14], salimabromides [15], salimyxins and enhygrolides [16].

**Figure 1:**
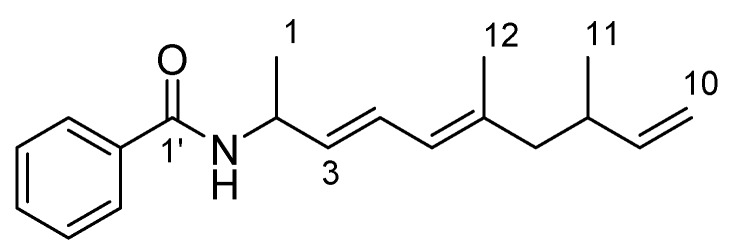
Structure of haliamide (**1**).

*Haliangium ochraceum* SMP-2 is the first marine myxobacterium, from which we discovered a potent antifungal agent haliangicin [10,11]. During our ongoing investigation on the secondary metabolome of *H. ochraceum*, a new PKS-NRPS hybrid compound, haliamide (**1**), was isolated and identified (Figure 1). In this paper, we report the structural elucidation, bioactivity and the decipherment of the biosynthetic system of haliamide (**1**).

## 2. Results and Discussion

### 2.1. Isolation and Structural Elucidation

The marine myxobacterium *H. ochraceum* SMP-2 showed an optimal NaCl demand of 2% (*w*/*v*) for its growth and needs approximately two weeks for the maximal production of secondary metabolites. The bacterial cells from a total of 3.5 L cultures were extracted with methanol. After solvent partitioning of the methanol extract, a hexane–EtOAc (1:1) fraction was chromatographed on silica gel followed by reverse-phase HPLC to give 1.9 mg of haliamide (**1**) as well as haliangicin.

**Figure 2:**
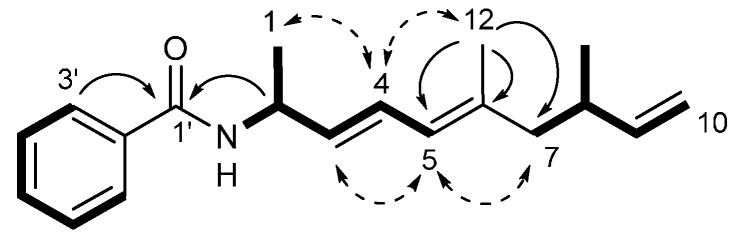
Key 2-demensional NMR correlations in haliamide (**1**). Bold lines: DQF-COSY, curve arrows: HMBC, dotted arrows: NOESY.

Haliamide (**1**) displayed the IR absorption bands at 3305, 1635 and 1536 cm^−1^, which indicated the presence of an amide group. Its molecular formula was determined to be C_19_H_25_NO by the high-resolution ESI-MS. The ^1^H- and ^13^C-NMR data of **1** in CDCl_3_ is summarized in Table 1. Three partial structures, C_6_H_5_–, –NH–CH(CH_3_)–CH=CH– and –CH_2_–CH(CH_3_)–CH=CH_2_, were deduced by DQF-COSY correlations (Figure 2), in which proton-carbon direct connectivity was determined by the heteronuclear single quantum coherence (HSQC) experiment. The remaining three quaternary carbons were assigned by the heteronuclear multiple bond correlation (HMBC) to the carbonyl at C-1′ (δ_C_ 166.5) and the two sp^2^ carbons at C-2′ (δ_C_ 134.8) and C-6 (δ_C_ 138.0) and connected to the above-mentioned partial structures (Figure 2). The geometry of the diene moiety was determined to be all-*E* based on the ^3^*J*_H3-H4_ value of 15.2 Hz and the NOE correlations of H-4/H-12 and H-5/H-7 (Figure 2). The benzamide structure was also supported by the absorption maximum at 242 nm.

**Table 1:** NMR data for haliamide (**1**) in CDCl_3_.

Position	δ_C_	δ_H_ mult. (*J* in Hz)
1	20.8	1.38 d (6.8)
2	47.0	4.84 ddq (6.0, 7.2, 6.8)
3	131.9	5.63 dd (6.0, 15.2)
4	126.8	6.46 dd (10.8, 15.2)
5	125.5	5.81 d (10.8)
6	138.0	-
7	47.4	1.97 dd (7.6, 13.6), 2.10 dd (7.2, 13.6)
8	35.6	2.36 dddq (6.8, 7.2, 7.6, 6.4)
9	144.2	5.73 ddd (6.8, 10.4, 17.2)
10	112.4	4.91 d (10.4), 4.96 d (17.2)
11	19.5	0.95 d (6.4)
12	16.7	1.73 s
1′	166.5	-
2′	134.8	-
3′, 7′	126.8	7.77 d (7.5)
4′,6′	128.5	7.43 t (7.5)
5′	131.4	7.49 t (7.5)
NH	-	6.04 d (7.2)

Measured at 400 MHz for ^1^H and 100 MHz for ^13^C.

### 2.2. Identification of the Biosynthetic Precursors

**Figure 3:**
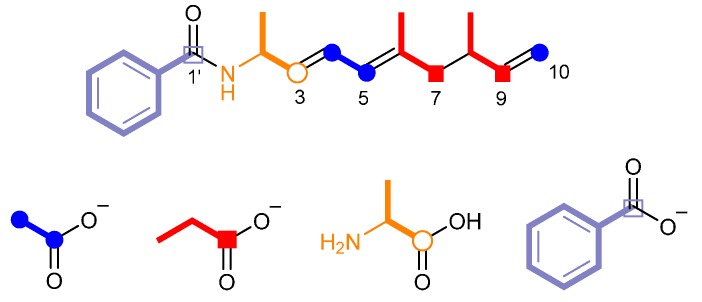
Biosynthetic building blocks of haliamide (**1**) deduced from feeding experiments.

To obtain supporting information for analyzing the haliamide biosynthetic machinery, we carried out feeding experiments with stable-isotope labeled compounds that were plausible as biosynthetic building blocks (Supplementary Materials, Figure S1). The addition of sodium [1,2-^13^C_2_]acetate to the culture medium of *H. ochraceum* led to the enhancement of ^13^C-NMR signals of C-4 (δ_C_ 126.8, d, *J* = 56 Hz), C-5 (δ_C_ 125.5, d, *J* = 56 Hz) and C-10 (δ_C_ 112.4, s), corresponding one intact acetate unit and one acetate-derived terminal methylene in the haliamide structure (Figure 3). The second feeding experiment with sodium [1-^13^C]propionate gave two isotope-labeled carbons of C-7 (δ_C_ 47.4) and C-9 (δ_C_ 144.2). Therefore, the polyene chain in haliamide is derived from two acetate and two propionate units (Figure 3). The third feeding experiment with dl-[1-^13^C]alanine enriched ^13^C nucleus at C-3. Finally, the origin of the benzoyl moiety was clarified to be benzoic acid because the carbonyl carbon of [1-^13^C]benzoic acid was incorporated into the C-1′ position in **1** (Figure 3). Since [1,2-^13^C_2_] acetate was not incorporated into the benzoyl group, the benzoate unit may be derived via shikimate pathway but not via polyketide one. The benzoate unit as a biosynthetic precursor is not very common and has been reported for a few natural products such as soraphen A [17] and enterocin [18].

### 2.3. Proposed Biosynthetic Mechanism of Haliamide *(**1**)*

**Figure 4:**
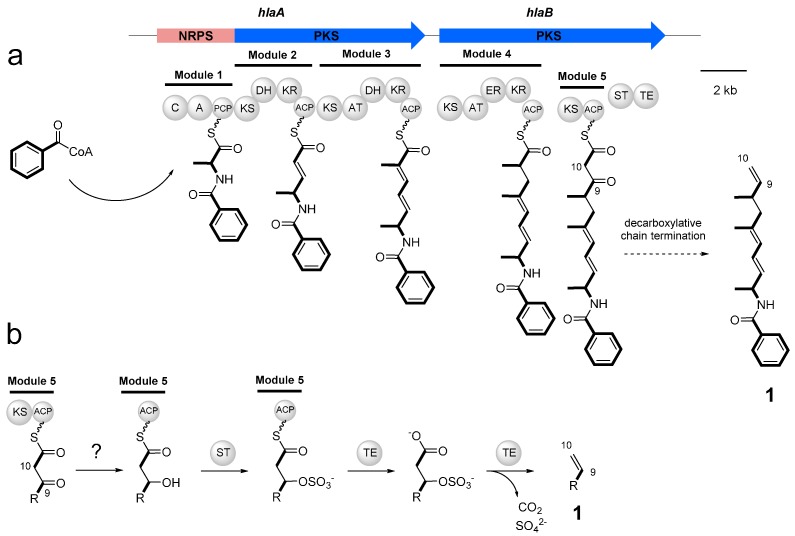
Proposed biosynthetic machinery of haliamide (**1**). (**a**) Genetic organization of the haliamide biosynthetic gene cluster (*hla*, 21.7 kbp) and a biosynthetic pathway for **1**; (**b**) Decarboxylative chain termination at the final step leading to the formation of the terminal olefin at C-9 and C-10. Abbreviations: A, adenylation domain; ACP, acyl carrier protein; AT, acyl transferase; C, condensation domain; DH, dehydratase; ER, enoyl reductase; KR, ketoreductase; KS, ketosynthase; PCP, peptide carrier protein; ST, sulfotransferase; TE, thioesterase.

The chemical structure of haliamide (**1**) and its biosynthetic precursors described above suggest it to be a hybrid compound biosynthesized by PKS and NRPS. The *in silico* analysis of the genome data of *H. ochraceum* using antiSMASH [19] revealed a candidate gene cluster, which possesses one NRPS module followed by four PKS modules and is in a good agreement with the assembly of the biosynthetic units in **1** (Figure 3). The putative haliamide gene cluster (*hla*) consists of one NRPS/PKS hybrid gene (*hlaA*) and one PKS gene (*hlaB*), spanning a region of 21.7 kbp (Figure 4a). The biosynthesis of haliamide (**1**) was proposed to begin with benzoyl CoA. Although the loading mechanism of this starter remains unclear due to the absence of the loading module in the *hla* gene cluster, the direct condensation of the benzoyl CoA starter and the forthcoming alanine unit on module 1 could be possible as a similar starter loading mechanism was recently reported in the biosynthesis of macyranones [20]. The C domain in HlaA may catalyze the formation of the amide bond. The subsequent chain elongation reactions take place by four PKS modules (Module 2–5), which incorporate two malonyl-CoAs and two methylmalonyl-CoAs to construct the polyketide backbone of **1** (Figure 4a). There are some unusual features in the PKS modules. First, there are some missing domains, including AT domains in modules 2 and 5, DH domain in module 3 and KR domain in module 5 (Supplementary Materials, Figure S2). The function of these missing domains may be complemented by some trans-acting enzymes. For the missing AT domains, a standalone acyltransferase (Hoch_5652) was found in the genome of *H. ochraceum*, which harbors the APFH motif that suggests this enzyme to be malonate-specific. Such substrate specificity well fits for the malonate incorporation by modules 2 and 5, as demonstrated by the feeding experiments (Figure 3). On the other hand, no candidates of trans-acting DH and KR domains were found. We anticipated that some oxidoreductases downstream to the hla cluster, e.g., cytochrome P450 (Hoch_0804, Hoch_0812, Hoch_0813 and Hoch_0814) and amine oxidase (Hoch_0806), may complement these missing domains, though the real roles of these proteins remain unclear. Second, the AT domain of module 4 indicates a malonate (acetate unit)-specific conserved motif HAFH [21] (Supplementary Materials, Figure S3) despite the fact that this module incorporated one methylmalonate (propionate) unit as shown in the feeding experiments. Such inconsistencies are not common in the typical bacterial PKS biosynthesis but sometimes observed in myxobacteria. We noticed that the AT domain of module 4 is phylogenetically close to the AT domain of EpoC (Supplementary Materials, Figure S4), a promiscuous AT loading both malonate and methylmalonate in the epothilone biosynthetic pathway from terrestrial myxobacterium *Sorangium cellulosum* [22].

After integrating the last biosynthetic block (acetate unit) by module 5, the biosynthesis undergoes a particular chain termination by decarboxylation, leading to the formation of a terminal olefin of the haliamide molecule, which may be mediated by the sulfotransferase (ST)-thioesterase (TE) domains encoded in the C-terminus of HlaB (Figure 4b). These domains are homologous to CurM TE and CurM ST (identity of 37% and 41%, respectively) of curacin A [23], in which β-keto group is reduced by KR domain and the resulting β-hydroxyl intermediate is sulfated by ST, followed by TE-catalyzed hydrolysis coupled with decarboxylative elimination to give a terminal olefin [24]. The haliamide biosynthesis was predicted to be terminated in a similar way, though a KR domain to generate β-hydroxyl group is absent in module 5 (Figure 4b).

### 2.4. Bioactivities of Haliamide *(**1**)*

The cytotoxic effect of the isolated compound haliamide (**1**) was evaluated by MTT assay using a tumor cell line (HeLa-S_3_). The IC_50_ was determined to be 12 μM (a positive control, paclitaxel: IC_50_ = 8.9 nM, Supplementary Materials, Figure S6). Haliamide (**1**) showed no inhibitory activity against the plant pathogen *Phytophthora capsici* (minimum inhibition dose: >30 μg/disk), whereas haliangicin isolated from the same myxobacterium showed an activity at 0.1 μg/disk or higher doses. In the other antimicrobial tests, **1** did not show inhibitory activities (MIC > 128 μg/mL) against a fungus (*Candida rugosa*), a gram negative (*Escherichia coli*) and a gram positive bacterium (*Bacillus subtilis*).

## 3. Materials and Methods

### 3.1. General

Flash chromatography was carried out with a medium-pressure gradient system equipped with a Pump Module C-605 and a Pump Manager C-615 (BÜCHI, Flawil, Switzerland). Preparative HPLC was performed on a high-pressure gradient system equipped with PU-2087 plus pumps and a UV-2075 plus detector (Jasco, Tokyo, Japan). Specific rotation was measured by using a DIP-370 digital polarimeter (Jasco). FT-IR and UV spectra were recorded on FT/IR-4100 and V-530 spectrometers (Jasco), respectively. Mass spectra (MS) were recorded on a Mariner Biospectrometry Workstation (Applied Biosystems of Thermo Fisher Scientific, Waltham, MA, USA) in the positive-ESI mode. ^1^H- and ^13^C-NMR spectra were recorded at 27 °C on an Avance 400 (400 MHz for ^1^H) or Avance III HD 600 Cryo-Probe (600 MHz for ^1^H) spectrometer (Bruker, Rheinstetten, Germany). Chemical shifts (ppm) were referenced to the solvent (CDCl_3_) peaks at δ_H_ 7.26 ppm (residual CHCl_3_) and δ_C_ 77.0 ppm.

### 3.2. Isolation

The marine myxobacterium *Haliangium ochraceum* SMP-2 was cultivated in 2 L flasks containing 500 mL of the production medium at 30 °C and 180 rpm for two weeks as previously reported [10]. The cells and resin were harvested from 3.5 L culture broth by filtration and extracted with methanol (300 mL, three times) at 30 °C for 60 min on a horizontal shaker (120 rpm).The combined methanolic extracts were concentrated *in vacuo*. The resulting crude extract (5.63 g) was further partitioned between water and hexane/EtOAc (1/1, *v*/*v*), and the organic layer were concentrated to yield an oily sample (435.7 mg). The crude oil was chromatographed on a HI-FLASH™ silica gel column (size L, 30 g, Yamazen Co., Osaka, Japan) with a linear gradient of 10%–50% EtOAc in hexane (60 min) at 10 mL/min. The fractions (32.5 mg) eluted with 21%–49% EtOAc in hexane were combined and subjected to preparative HPLC [Develosil ODS-HG-5 (20 × 250 mm, Nomura Chemical Ltd., Seto, Japan), 75% MeOH, 6 mL/min, monitored at 205 nm] to obtain haliamide (**1**, 1.9 mg, *t*_R_ = 56 min): colorless oil, ${\left[\alpha \right]}_{\text{D}}^{22}$ − 3 (*c* 0.12, MeOH); UV (MeOH) λ_max_ 242 (ε 18,000) nm; IR (film) *ν*_max_ 3305 (br), 3064, 3029, 1635, 1536, 964, 694 cm^−1^; ESI-MS *m/z* 284.20 [M + H]^+^, 306.19 [M + Na]^+^, 322.16 [M + K]^+^. HR ESI-MS calcd. for C_19_H_26_NO 284.2009 [M + H]^+^; found 284.1989.

### 3.3. Feeding Experiments with Stable Isotope Labeled Precursors

The stable isotope-labeled compounds used were sodium [1,2-^13^C_2_]acetate (Cambridge Isotope Laboratories, Tewksbury, MA, USA), sodium [1-^13^C]propionate (Sigma-Aldrich Co. St. Louis, MO, USA), dl-[1-^13^C]alanine (Isotec, Canton, GA, USA) and [1-^13^C]benzoic acid (Aldrich). *H. ochraceum* was cultured in the above-mentioned production medium (100 mL in six 500 mL flasks or 750 mL in one 2 L flask) in the presence of 2% (*w*/*v*) adsorbent resin (SEPABEADS SP207, Mistubishi Chemical Co., Tokyo, Japan). A filter-sterile solution of an isotope-labeled compound (sodium [1,2-^13^C_2_]acetate, sodium [1-^13^C]propionate, dl-[1-^13^C]alanine in water, or [1-^13^C]benzoic acid neutralized with 6 N NaOH and diluted with water, 200 mM) was added into 4-days bacterial culture to the final concentration of 4 mM. After two weeks cultivation in total, the cells and the resin were collected and extracted with methanol (300 mL, three times). The combined methanolic extracts were concentrated and then partitioned between water (200 mL) and EtOAc (200 mL). The ethyl acetate part was dissolved in 50% MeOH (50 mL) and extracted twice with hexane–EtOAc (4:1, 50 mL). The crude oil obtained from the upper layer was chromatographed on a HI-FLASH™ silica gel column (size S, flow rate: 3 mL/min) with a linear gradient using 10%–50% EtOAc in hexane (30 min). The haliamide-containing fractions were combined and then subjected to preparative HPLC [Develosil ODS-HG-5 (10 × 250 mm, Nomura Chemical Ltd.), 60% MeCN, 3 mL/min, monitored at 205 nm] to afford [1,2-^13^C_2_]acetate-labeled haliamide (0.3 mg), [1-^13^C]propionate-labeled haliamide (0.5 mg), [1-^13^C]alanine-labeled haliamide (0.5 mg), and [1-^13^C]benzoic acid-labeled haliamide (0.2 mg), respectively.

### 3.4. Analysis of the Haliamide Biosynthetic Gene Cluster (hla)

The genome data of *H. ochraceum* SMP-2T (Accession No. NC_013440.1 [25]) was analyzed by antiSMASH [19]. The sequence for the putative biosynthetic gene cluster *hla* was annotated by BLAST and CDD [26]. The multiple alignments of amino acid sequences were generated by Clustal Omega program provided by EMBL.

### 3.5. Bioassay

#### 3.5.1. Cytotoxicity Assay

HeLa-S3 (SC) cells were provided by the RIKEN BRC through the National Bio-Resource Project of the MEXT, Japan. The cells were cultured in Eagle’s minimal essential medium (EMEM) (Wako Pure Chemical Industries, Osaka, Japan) supplemented with 10% bovine serum, 100 units/mL penicillin, and 100 μg/mL streptomycin (Thermo Fisher Scientific Inc., Waltham, MA, USA). A total of 10,000 cultured cells were seeded into each well of a 96-well plate containing 99 μL of the same medium. After preincubation for 24 h at 37 °C in an atmosphere of 5% CO_2_, a compound (haliamide or paclitaxel as a positive control) in 1 μL of dimethyl sulfoxide (DMSO), or only 1 μL of DMSO as control, was added to each well, and the cells were incubated an additional 48 h. A solution (10 μL) of 3-(4,5-dimethylthiazol-2-yl)-2,5-diphenyltetrazolium bromide (MTT) in phosphate buffer saline (PBS) (5 mg/mL) was then added to each well, and the plate was incubated for an additional 3 h. Subsequently, the medium was removed by aspiration, any generated formazan was dissolved in 100 μL of DMSO, and the absorbance was measured at 595 nm using a Multiskan FC microplate reader (Thermo Fisher Scientific). Values are means ± standard error (*n* = 4).

#### 3.5.2. Anti-Oomycete Activity

The phytopathogenic oomycete *Phytophthora capsici* NBRC 30696, purchased from NITE-Biological Resource Center (NBRC, Chiba, Japan), was cultured on a potato-agar medium (in 1 L, potato broth from 200 g of fresh potato, glucose (20 g), and agar (20 g)) in a 9-cm dish at 25 °C for 7 days in the dark. A piece of the colony was then inoculated on the center of a 5% V8-agar medium (V8 vegetable juice (5 mL), agar (1.5 g), and water (95 mL)) in a 9-cm dish and incubated at 25 °C for 48 h in the dark until the colony grew to approximately 4 cm in diameter. A paper disc (6 mm in diameter) impregnated with a sample was placed 1 cm away from the front of the colony. After incubating for 22–24 h, the distance between the edge of the colony and the paper disc (control: 0 mm) was measured. The activity was defined as a minimum dose that induced a definite distance (0.5 mm or wider) between the colony and the disk. The tested doses were 1, 3, 10, and 30 μg/disk.

#### 3.5.3. MIC Assay

The microorganisms, *Candida rugosa* AJ 14513, *Bacillus subtilis* AJ 12865, and *Escherichia coli* AJ 3837, were provided by the Institute for Innovation, Ajinomoto Co., Inc. (Kanagawa, Japan). The protocols used for the assay are in the followings. For *B. subtilis* and *E. coli*, see *Methods for Dilution Antimicrobial Susceptibility Tests for Bacteria That Grow Aerobically; Approved Standard*—Ninth Edition. CLSI document M07-A9 (ISBN 1-56238-783-9), Clinical and Laboratory Standards Institute (Wayne, PA, USA), 2012. For *C. rugosa*, see *Reference Method for Broth Dilution Antifungal Susceptibility Testing of Yeasts; Approved Standard*—Second Edition. NCCLS document M27-A2 (ISBN 1-56238-469-4), NCCLS (Wayne, PA, USA), 2002. The tested concentrations were 1, 2, 4, 8, 16, 32, 64, and 128 μg/mL.

## 4. Conclusions

In the present study, we discovered a new PKS-NRPS hybrid type metabolite, haliamide (**1**), from the halophilic myxobacterium *H. ochraceum* and evaluated its biological activities. The identification of the biosynthetic units for **1** and the genome mining of its producer provided a putative biosynthetic gene cluster (*hla*). Further, genomic analysis suggests that at least five more PKS-NRPS gene clusters are harbored in the genome of *H. ochraceum*, which prompts us to discover additional unknown metabolites of this hard-to-culture marine myxobacterium.

## Supplementary Materials

Supplementary materials can be accessed at: http://www.mdpi.com/1420-3049/21/1/59/s1.

## References

[1] Weissman K.J., Mueller R. (2009). A brief tour of myxobacterial secondary metabolism. Bioorg. Med. Chem..

[2] Reichenbach H. (2001). Myxobacteria, producers of novel bioactive substances. J. Ind. Microbiol. Biotechnol..

[3] Wenzel S.C., Muller R. (2009). Myxobacteria—“Microbial factories” for the production of bioactive secondary metabolites. Mol. Biosyst..

[4] Iizuka T., Jojima Y., Fudou R., Tokura M., Hiraishi A., Yamanaka S. (2003). *Enhygromyxa salina* gen. nov., sp nov., a slightly halophilic myxobacterium isolated from the coastal areas of Japan. Syst. Appl. Microbiol..

[5] Schaeberle T.F., Goralski E., Neu E., Erol O., Hoelzl G., Doermann P., Bierbaum G., Koenig G.M. (2010). Marine Myxobacteria as a Source of Antibiotics—Comparison of Physiology, Polyketide-Type Genes and Antibiotic Production of Three New Isolates of *Enhygromyxa salina*. Mar. Drugs.

[6] Iizuka T., Jojima Y., Fudou R., Hiraishi A., Ahn J.W., Yamanaka S. (2003). *Plesiocystis pacifica* gen. nov., sp nov., a marine myxobacterium that contains dihydrogenated menaquinone, isolated from the Pacific coasts of Japan. Int. J. Syst. Evol. Microbiol..

[7] Iizuka T., Jojima Y., Hayakawa A., Fujii T., Yamanaka S., Fudou R. (2013). *Pseudenhygromyxa salsuginis* gen. nov., sp nov., a myxobacterium isolated from an estuarine marsh. Int. J. Syst. Evol. Microbiol..

[8] Still P.C., Johnson T.A., Theodore C.M., Loveridge S.T., Crews P. (2014). Scrutinizing the Scaffolds of Marine Biosynthetics from Different Source Organisms: Gram-Negative Cultured Bacterial Products Enter Center Stage. J. Nat. Prod..

[9] Komaki H., Fudou R., Iizuka T., Nakajima D., Okazaki K., Shibata D., Ojika M., Harayama S. (2008). PCR Detection of Type I Polyketide Synthase Genes in Myxobacteria. Appl. Environ. Microbiol..

[10] Fudou R., Iizuka T., Yamanaka S. (2001). Haliangicin, a novel antifungal metabolite produced by a marine myxobacterium 1. Fermentation and biological characteristics. J. Antibiot..

[11] Fudou R., Iizuka T., Sato S., Ando T., Shimba N., Yamanaka S. (2001). Haliangicin, a novel antifungal metabolite produced by a marine myxobacterium 2. Isolation and structural elucidation. J. Antibiot..

[12] Kundim B.A., Itou Y., Sakagami Y., Fudou R., Iizuka T., Yamanaka S., Ojika M. (2003). New haliangicin isomers, potent antifungal metabolites produced by a marine myxobacterium. J. Antibiot..

[13] Ojika M., Inukai Y., Kito Y., Hirata M., Iizuka T., Fudou R. (2008). Miuraenamides: Antimicrobial cyclic depsipeptides isolated from a rare and slightly halophilic myxobacterium. Chem. Asian J..

[14] Iizuka T., Fudou R., Jojima Y., Ogawa S., Yamanaka S., Inukai Y., Ojika M. (2006). Miuraenamides A and B, novel antimicrobial cyclic depsipeptides from a new slightly halophilic myxobacterium: Taxonomy, production, and biological properties. J. Antibiot..

[15] Felder S., Dreisigacker S., Kehraus S., Neu E., Bierbaum G., Wright P.R., Menche D., Schaberle T.F., Konig G.M. (2013). Salimabromide: Unexpected Chemistry from the Obligate Marine Myxobacterium *Enhygromyxa salina*. Chem. Eur. J..

[16] Felder S., Kehraus S., Neu E., Bierbaum G., Schaberle T.F., Konig G.M. (2013). Salimyxins and Enhygrolides: Antibiotic, Sponge-Related Metabolites from the Obligate Marine Myxobacterium *Enhygromyxa salina*. ChemBioChem.

[17] Ligon J., Hill S., Beck J., Zirkle R., Molnar I., Zawodny J., Money S., Schupp T. (2002). Characterization of the biosynthetic gene cluster for the antifungal polyketide soraphen A from *Sorangium cellulosum* So ce26. Gene.

[18] Kalaitzis J.A., Cheng Q., Meluzzi D., Xiang L.K., Izumikawa M., Dorrestein P.C., Moore B.S. (2011). Policing starter unit selection of the enterocin type II polyketide synthase by the type II thioesterase EncL. Bioorg. Med. Chem..

[19] Blin K., Medema M.H., Kazempour D., Fischbach M.A., Breitling R., Takano E., Weber T. (2013). antiSMASH 2.0-a versatile platform for genome mining of secondary metabolite producers. Nucleic Acids Res..

[20] Keller L., Plaza A., Dubiella C., Groll M., Kaiser M., Muller R. (2015). Macyranones: Structure, Biosynthesis, and Binding Mode of an Unprecedented Epoxyketone that Targets the 20S Proteasome. J. Am. Chem. Soc..

[21] Yadav G., Gokhale R.S., Mohanty B. (2003). Computational approach for prediction of domain organization and substrate specificity of modular polyketide synthases. J. Mol. Biol..

[22] Petkovic H., Sandmann A., Challis L.R., Hecht H.J., Silakowski B., Low L., Beeston N., Kuscer E., Garcia-Bernardo J., Leadlay P.F. (2008). Substrate specificity of the acyl transferase domains of EpoC from the epothilone polyketide synthase. Org. Biomol. Chem..

[23] Chang Z.X., Sitachitta N., Rossi J.V., Roberts M.A., Flatt P.M., Jia J.Y., Sherman D.H., Gerwick W.H. (2004). Biosynthetic pathway and gene cluster analysis of curacin A, an antitubulin natural product from the tropical marine cyanobacterium *Lyngbya majuscula*. J. Nat. Prod..

[24] Gu L.C., Wang B., Kulkarni A., Gehret J.J., Lloyd K.R., Gerwick L., Gerwick W.H., Wipf P., Hakansson K., Smith J.L. (2009). Polyketide Decarboxylative Chain Termination Preceded by *O*-Sulfonation in Curacin A Biosynthesis. J. Am. Chem. Soc..

[25] Ivanova N., Daum C., Lang E., Abt B., Kopitz M., Saunders E., Lapidus A., Lucas S., del Rio T.G., Nolan M. (2010). Complete genome sequence of *Haliangium ochraceum* type strain (SMP-2(T)). Stand. Genom. Sci..

[26] Marchler-Bauer A., Lu S.N., Anderson J.B., Chitsaz F., Derbyshire M.K., de Weese-Scott C., Fong J.H., Geer L.Y., Geer R.C., Gonzales N.R. (2011). CDD: A Conserved Domain Database for the functional annotation of proteins. Nucleic Acids Res..

