# Supplementary material for: Isolation and Biosynthetic Analysis of Haliamide, a New PKS-NRPS Hybrid Metabolite from the Marine Myxobacterium Haliangium ochraceum
Source: Molecules. 2016 Jan 6;21(1):59. doi: 10.3390/molecules21010059 (PMC6274090; doi:10.3390/molecules21010059)
Supplement: Supplementary file 1 [file molecules-21-00059-s001.pdf]

Supplementary Materials: Isolation and Biosynthetic Analysis of Haliamide, a New PKS-NRPS Hybrid Metabolite from the Marine Myxobacterium *Haliangium ochraceum*

Yuwei Sun [1], Tomohiko Tomura [1], Junichi Sato [1], Takashi Iizuka [2], Ryosuke Fudou [3] and Makoto Ojika [1,*]

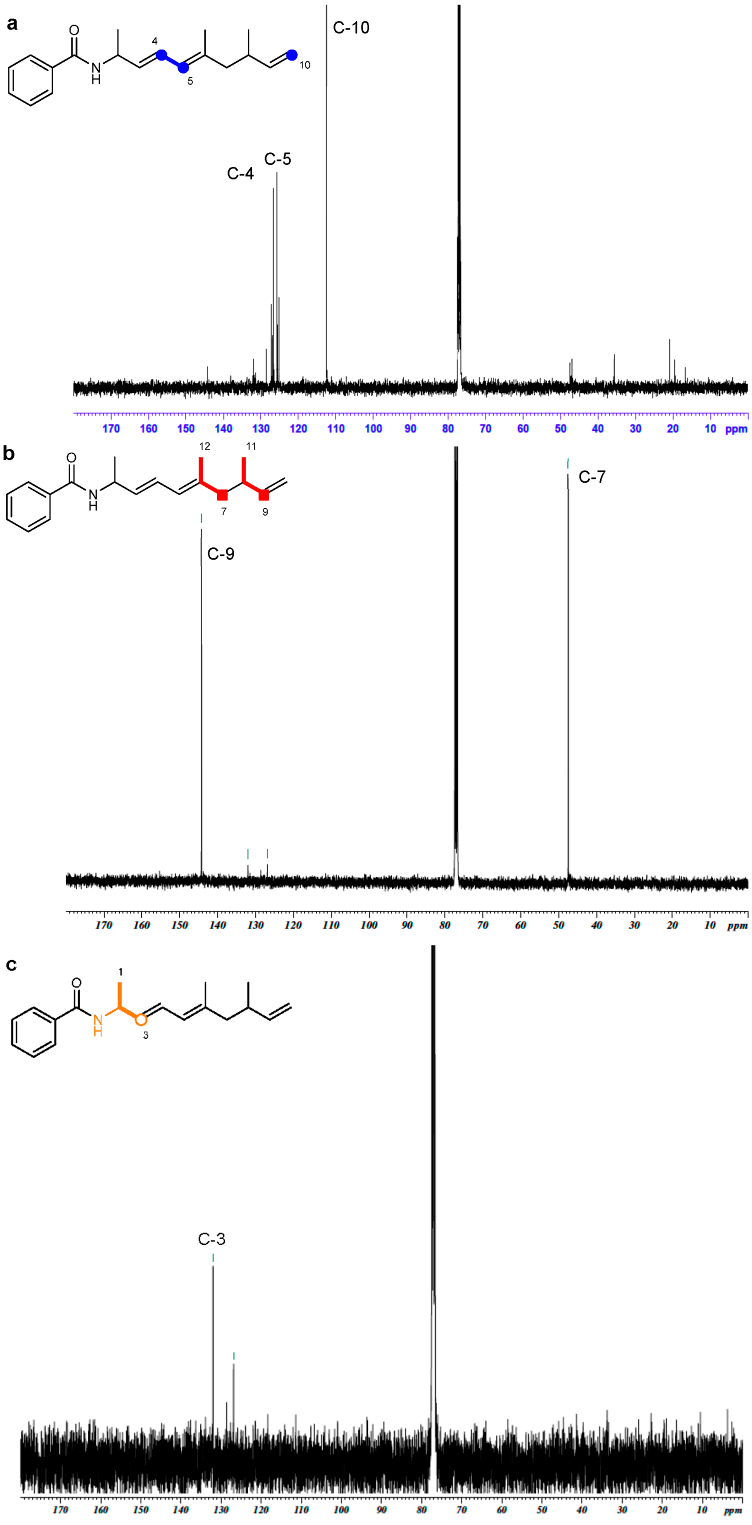

Figure S1. *Cont.*

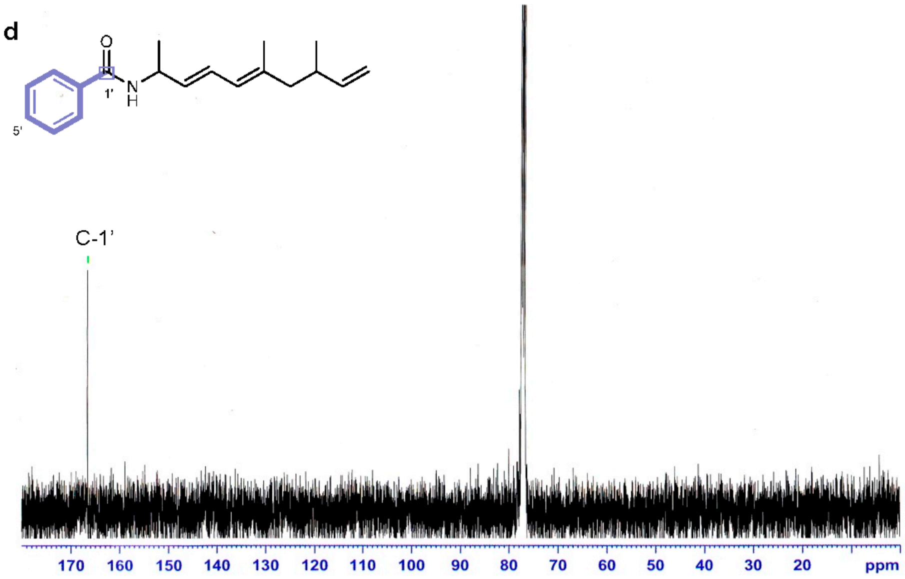

Figure S1. Feeding experiments with stable isotope labeled precursors. (**a**) ^{13}C-NMR spectrum (100 MHz) of [1,2-$^{13}C_2$]acetate-labeled haliamide in $CDCl_3$; (**b**) ^{13}C-NMR spectrum (100 MHz) of [1-^{13}C]propionate-labeled haliamide in $CDCl_3$; (**c**) ^{13}C-NMR spectrum (100 MHz) of DL-[1-^{13}C]alanine-labeled haliamide in $CDCl_3$; (**d**) ^{13}C-NMR spectrum (100 MHz) of [1-^{13}C]benzoic acid-labeled haliamide in $CDCl_3$.

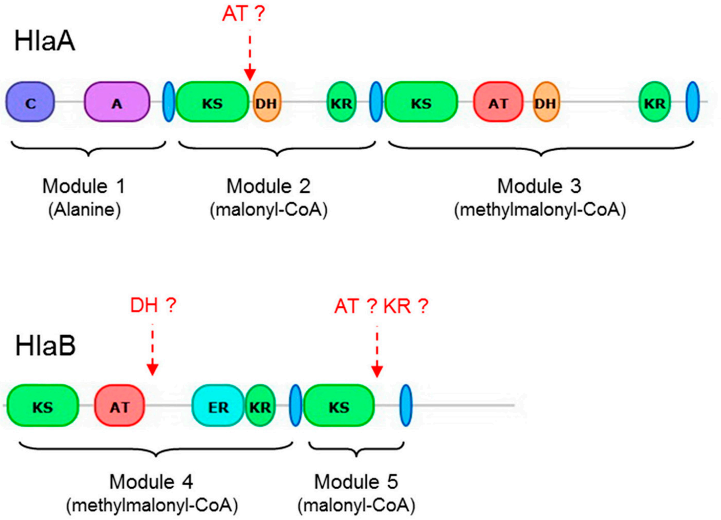

Gene	Protein	Length (aa)	Type	Domain position
hlaA	HlaA	4183	NRPS-type I PKS	C (11-293), A (477-861), PCP (944-1009), KS_1 (1023-1448), DH_1 (1482-1641), KR_1 (1923-2086), ACP_1 (2175-2245), KS_2 (2266-2690), AT (2793-3081), DH_2 (3148-3299), KR_2 (3779-3956), ACP_2 (4057-4128)
hlaB	HlaB	3045	Type I PKS	KS_1 (31-447), AT (550-840), ER (1131-1433), KR (1447-1616), ACP_1 (1709-1775), KS_2 (1793-2205), ACP_2 (2366-2430)

Figure S2. Detailed annotation of PKS and NRPS by antiSMASH 3.0 analysis. The annotation of HlaA and HlaB revealed 5 modules (1 NRPS and 4 PKSs), while several domains of PKSs are thought to be missing considering the structure of haliamide (**1**). These domains are AT domains in module 2 and 5, DH in module 4 and KR domain in module 5.

> HlaA_AT (module 3)

FPGQGAQWAGMARELYAREPAFRDALKACDRAIRDEAEWSLIAWLHGEGEAERIDRIQPALFAV
MVSLAGLWRDWGYEPAEVVGHSQGEVAAAYVAGALSLEDAVAIIVRRSAMLRTLSGRGAMMVV
ELTADKAAERIESVRDRVAVAVVNGPRSVVLSGDVEALETLGAELEAEGVYQRFVKVDVASHSPQ
MDPIRAKLLGALSEIAPQRGTTPIRSTVSTRTISGEEMDADYWWSNLRRPVRFGAVVEAMAQER
DILFLEISAHPLLRPAVEEQAPGRAVSSLRR

(underline: methylmalonate-specific motif)

> HlaB_AT (module 4)

LFTGQGAQRADMGRGLYEHHPGFRETLDRCADALGRAHDLREVMWSSDGRLDRTGWTQPALF
ALEVSLAALWRQWGIEPEVLVGHSVGEIAAACVAGVFSIEDGMRLVEARARLMDALPEGGAMVA
VRGQPARIERAVASAEGVSVAAFNGPDQVVISGASDAVQALASELAEAGLRAKALTVSHAFHSEL
MEPMLEDFRAALRDIRFHPPELPLVSNLRGELAGPEVASADYWVEHVRAPVRFLEGMRAAHAVG
VDHYLEIGPQPVLCRLGATCVPAGGGETWLPSLQR

(underline: malonate-specific motif)

> Hoch_5652 (possible *trans*-AT)

MGTALLFPGQGSQKVGMGKALVDAFPAARAVFDEADEALGFAISEVCFEGPADKLMLTAYSQPAI
LTQSIAVLRAAQAEGRIAQDGEVVAAMGHSLGEFTALVAAGAFTLSDAVRLVHLRGQAMQDAVPM
GEGGMAALLGLDAEAVQALCDEVAEGQVCVPANLNGAGQVVISGHAGAIERAVAAAKGKGAKR
AIKLQVSAPFHSPLMQPAAERLAEALDGIAIEPLRVPVISNVEAAPNSDAGRVKELLVAQVTGAVR
WEESMHALAAMDVSQGFEFGAGKVLRGLFSRTVKELPVHSLSEPDDIREGSNERGD

(underline: malonate-specific motif)

Figure S3. Sequence of AT domains.

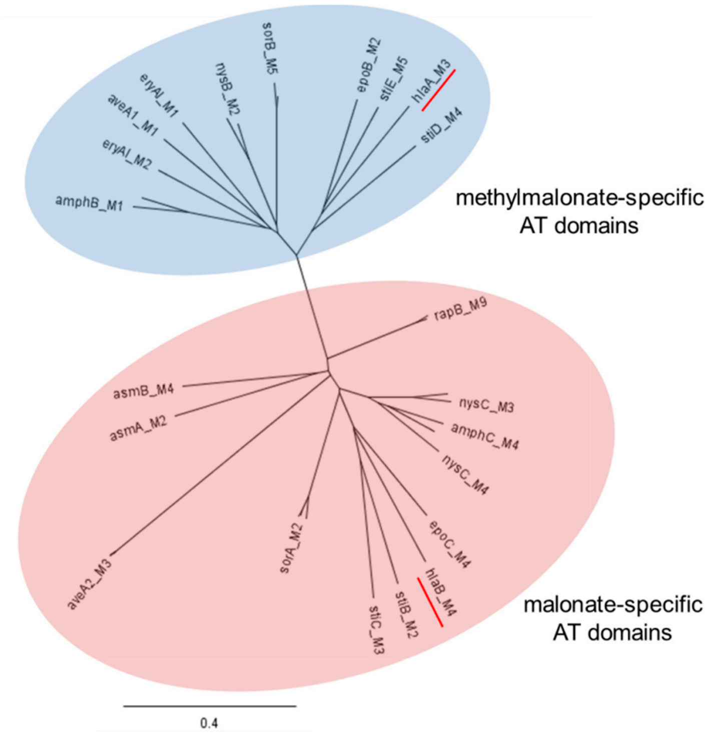

Figure S4. Phylogenetic tree of AT domains of type I PKS from several bacterial metabolites. Thirty three sequences from actinobacteria and proteobacteria were used, including AT domains in the amphotericin B (*amph*), ansamitocin (*asm*), avermectin (*ave*), epothilone (*epo*), erythromycin (*ery*), haliamide (*hla*), nystatin (*nys*), rapamycin (*rap*), soraphen (*sor*) and stigmatellin (*sti*) biosynthetic gene cluster. The AT domains from *hla* cluster are indicated by red underline. The AT domain of module 3 in hlaA is grouped in the methylmalonate specific clade, while AT domain of module 4 in hlaB is grouped in the malonate specific clade.

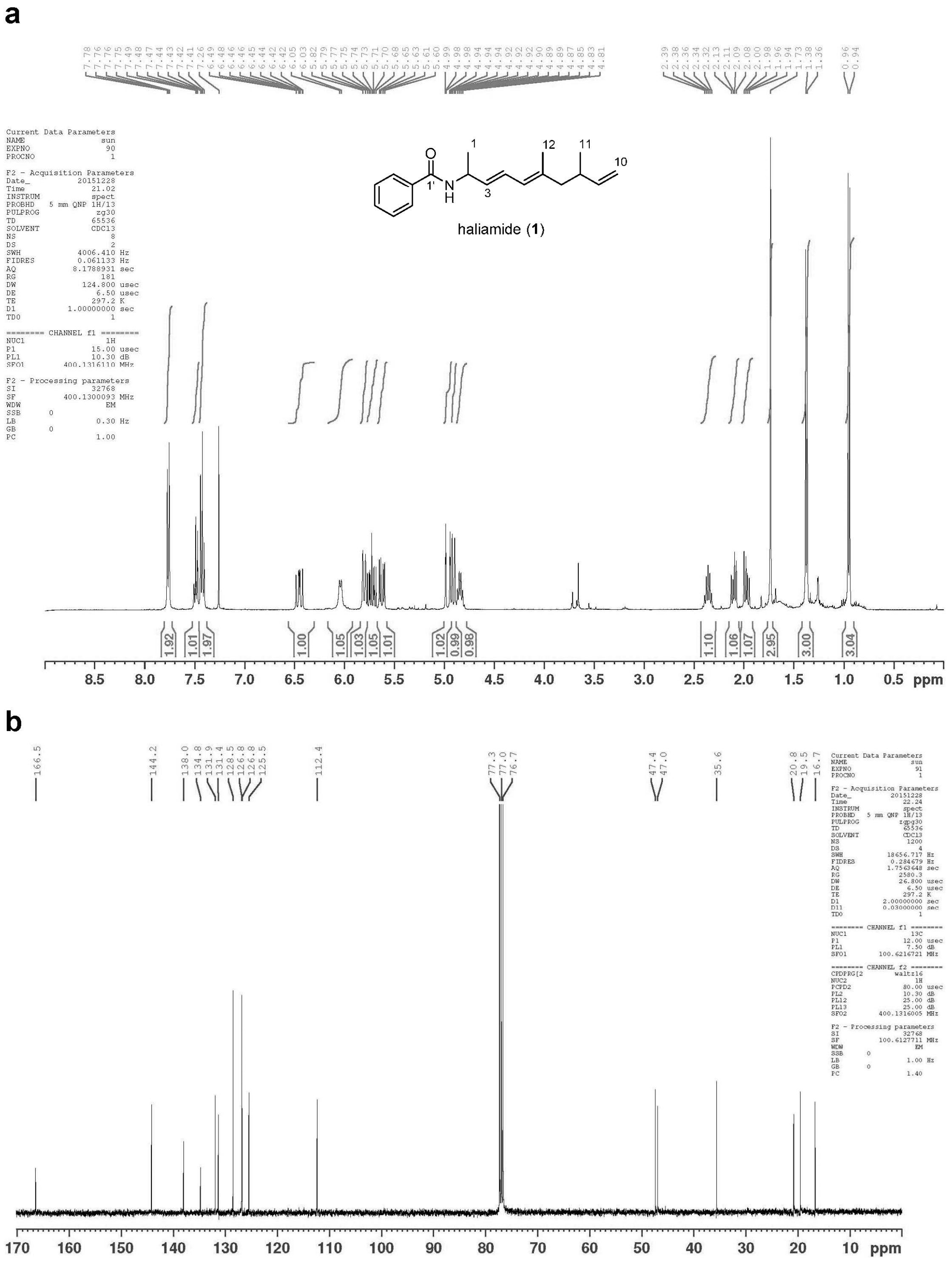

Figure S5. *Cont.*

c

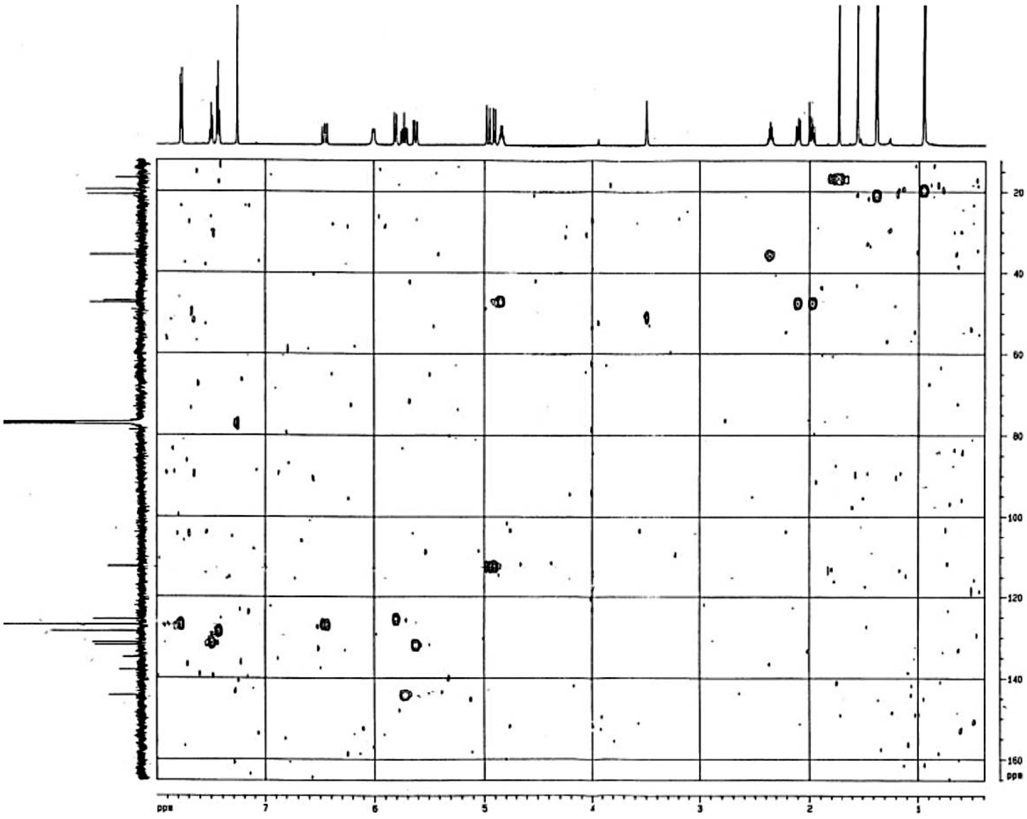

d

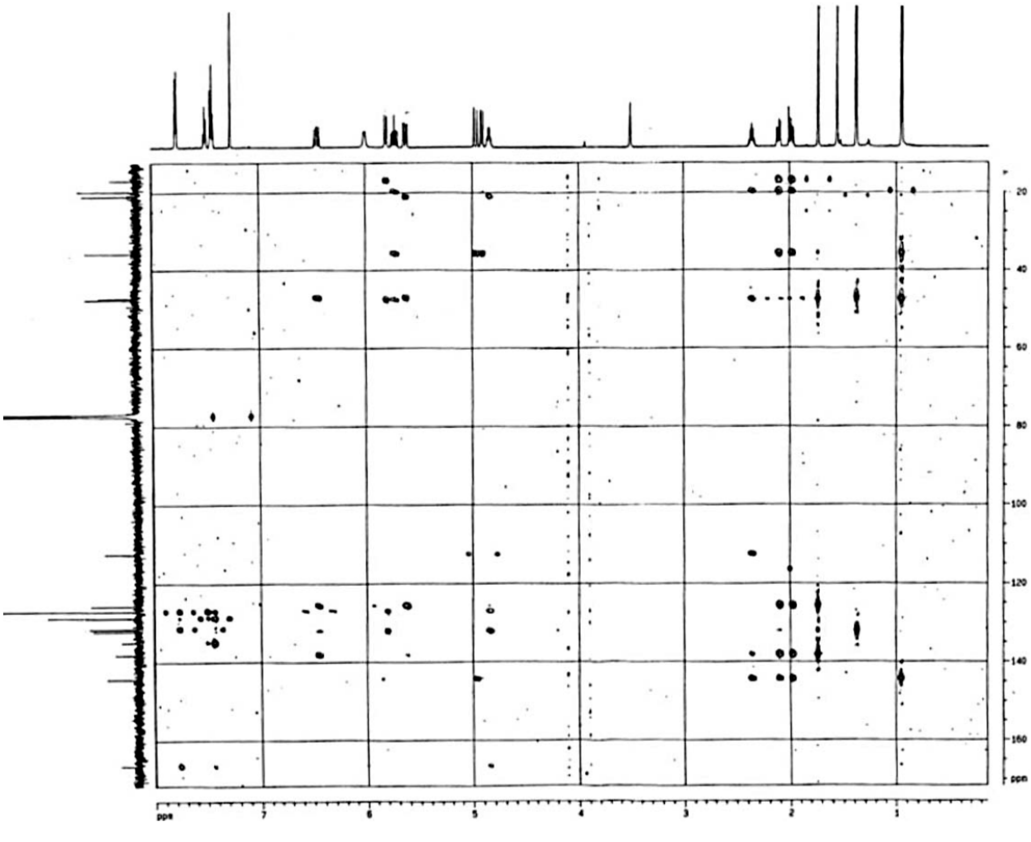

Figure S5. *Cont.*

e

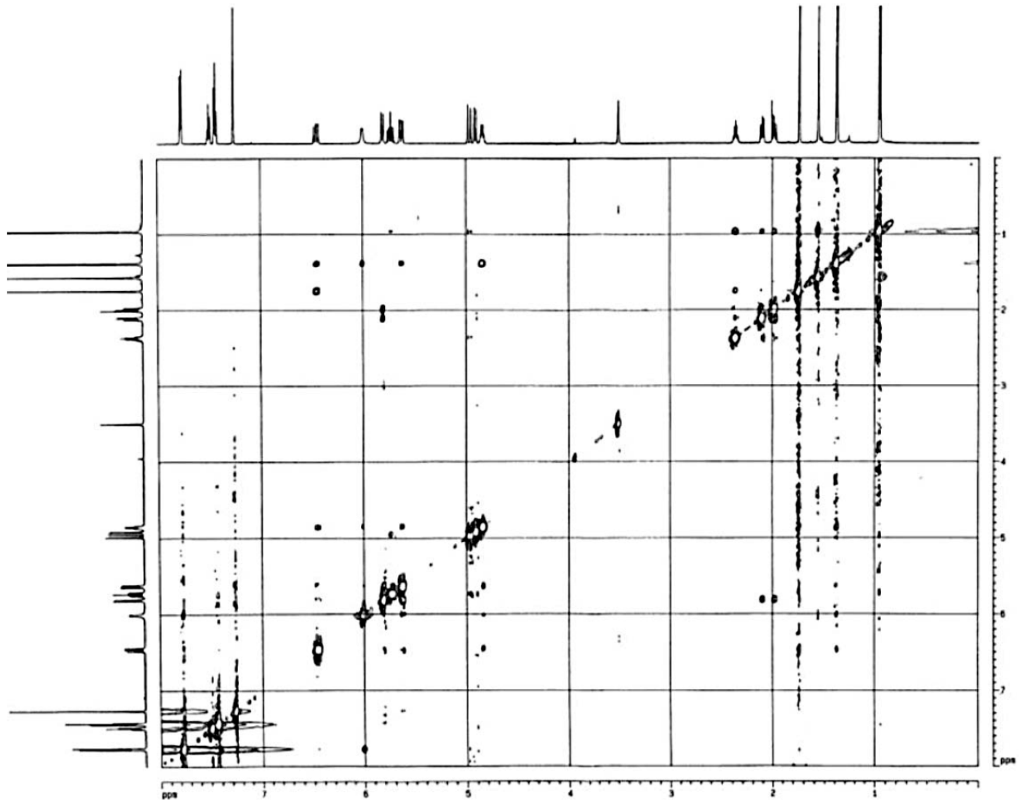

f

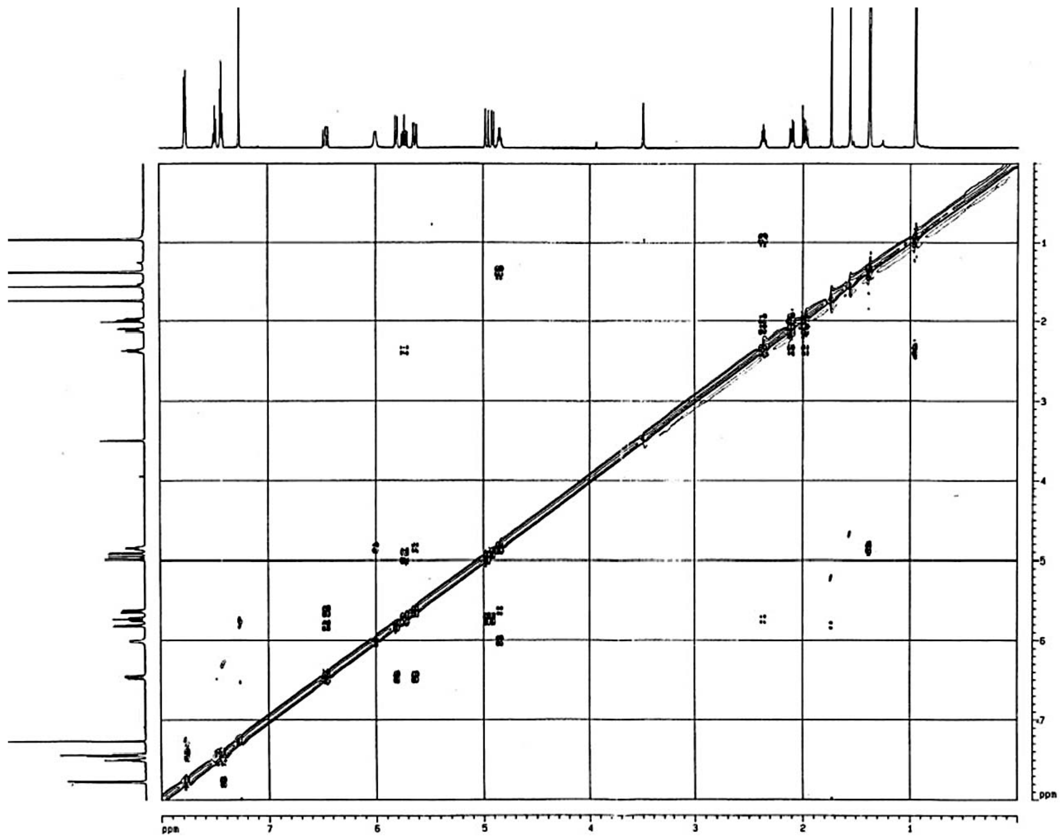

Figure S5. *Cont.*

g

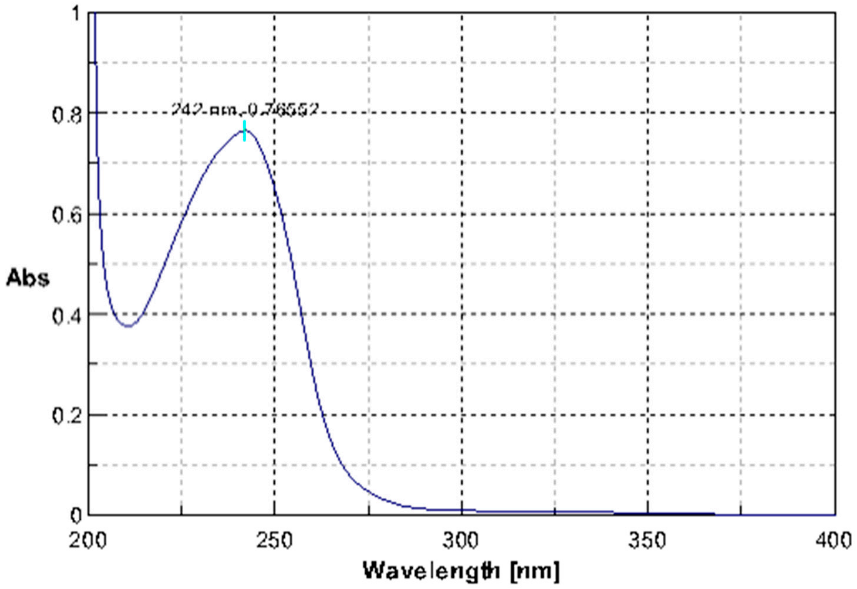

h

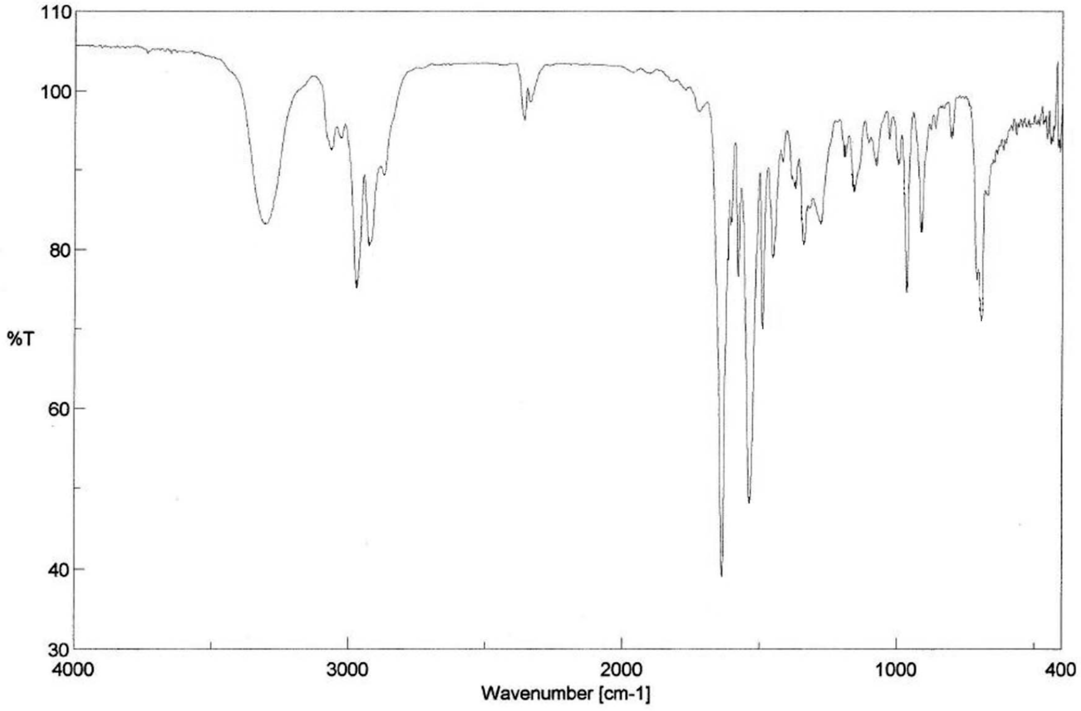

Figure S5. *Cont.*

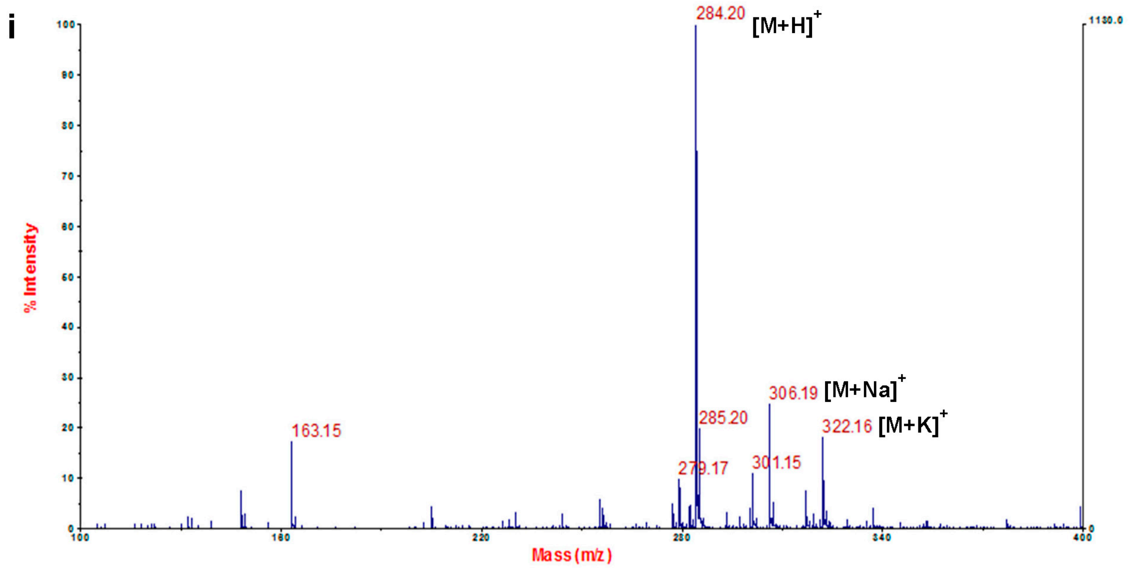

Figure S5. Spectra of haliamide (**1**). (**a**) ^{1}H-NMR (400 MHz, $CDCl_3$); (**b**) ^{13}C-NMR (100 MHz, $CDCl_3$); (**c**) HMQC; (**d**) HMBC; (**e**) NOESY; (**f**) DQF-COSY; (**g**) UV; (**h**) IR; (**i**) ESI-MS.

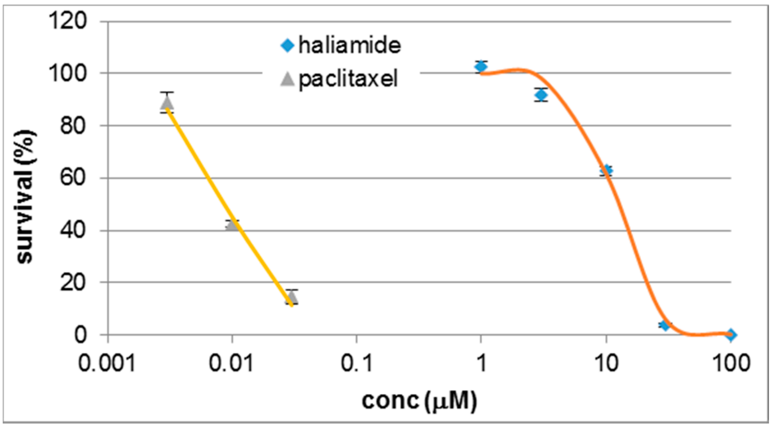

Figure S6. Cytotoxicity of haliamide (**1**) against HeLa-S3 cells. Markers indicate average values with SE (n = 4), and the lines indicate theoretical sigmoid curves ($y = 100/(1 + e^{-a(x-b)})$, x: log(μM)). Paclitaxel is a positive control (IC_{50} = 8.9 nM).
